# An Experimental Model of Primary Amoebic Meningoence phalitis Due to *Naegleria australiensis* in Iran

**Published:** 2018

**Authors:** Alireza LATIFI, Maryam NIYYATI, Seyyed Javad SEYYED TABAEI, Farid TAHVILDAR BIDEROUNI, Ali HAGHIGHI, Zohreh LASJERDI

**Affiliations:** Dept. of Medical Parasitology and Mycology, School of Medicine, Shahid Beheshti University of Medical Sciences, Tehran, Iran

**Keywords:** *Naegleria australiensis*, Experimental model, Iran

## Abstract

**Background:**

The main aim of the present research was to develop the experimental meningo encephalitis due to *Naegleria australiensis* isolated from geothermal water sources in mice model, November 2017 in Iran.

**Methods:**

*Naegleria australiensis* was isolated from geothermal water sources in northern Iran. The number of amoebae was adjusted to be 1×10^4^/ml amoebae. The experimental infection was done using 3 wk old male (BALB/c) mice. Seven animals were used for experimental amebic infection and one animal was selected for the control. Intranasal (IN) and intracerebral (IC) inoculation of amoebae were done. The mice were then monitored on daily observation and as soon as they present any brain involvement they sacrificed. The brain of all animals was then dislocated and passaged in non-nutrient agar.

**Results:**

One mouse out of seven infected mice were showed clinical symptoms of meningoencephalitis. Within few hours of culture of the brain, many vegetative forms of amoebae were detected in plate culture. The other infected animals and control mice showed no clinical symptoms until day 14. After 14 d all the animals sacrificed. The culture was negative up to one month.

**Conclusion:**

The lack of brain involvement of other animals in the present study could be due to animal immune system or it may be possible that the amoebae did not reach to olfactory bulb of nostrils.

## Introduction

Free-living amoebae include many genera such as Vahlkampfiids, *Acanthamoeba,* and *Balamuthia.* Among Vahlkampfiids some are of medical importance in human and animals such as *N. fowleri*, *N. australiensis, N. philippinensis and N. italica* (1). Until 1981, *Naegleria fowleri* was the only species among genus *Naegleria* that pathogenicity for humans had been confirmed. In 1981, during study of Pathogenicity of *N. fowleri*, explained pneumonitis induced by *N. fowleri* in mice (2). A new pathogenic species called *N. australiensis* was described (1). *N. fowleri* and *N. australiensis* may lead to sever and fatal disease called Primary meningoencephalitis (PAM) in human and animals, respectively. The disease is fulminant and acute and most of the patients would die. To date, *Paravahlkampfia francina* is also known as a cause of PAM (3).

In Iran some species of *Naegleria* including *N. pagei, N. fultoni, N. clarki, N. americana, N. dobsoni* and *N. polaris* has been described during last years (4–6). These species have been isolated from geothermal water sources of northern, north-west and Tehran recreational river sources in Iran. However, there is no report regarding the presence of *N. fowleri* in the region yet and more studies are needed to evaluate the environmental sources in the country. Regarding the PAM disease, there is only a single report of PAM in the country in a six-month-old infant. The disease was diagnosed by smear of cerebrospinal fluid (CSF) and the patient was treated successfully (7). In Iran, the most isolated *Naegleria* belonged to *N. australiensis* (8).

We aimed to develop the experimental meningoencephalitis due to *N. australiensis* isolated from geothermal water sources in mice model.

## Materials and Methods

*N. australiensis* (Accession number: KU380481) was isolated from Ramsar hot springs in northern Iran, December 2016 (8). This strain was isolated from a hot spring with temperature of 42 °C and pH of 5.7. The strain was kept on non-nutrient agar covered with a layer of *Escherchia coli*. Cloning of the strain was done in order to achieve a plate without any bacterial and fungi contamination. To this end, several passages of the strain were performed within 2 months. The plate surface was then covered with 5 ml of distilled Phosphate Buffer Saline (Ph: 7.2). After 5 min the amoebae on surface plate were isolated by cell scraper. The number of amoebae was then adjusted to be 1×10^4^ /ml amoebae.

The experimental infection was done using 3 wk old male (BALB/c) mice. The animals were 20–25 gr. seven animals were used for experimental amebic infection and one animal was selected as control. All the mice were male and inoculation was carried out at the same time and condition. Intranasal (IN) and intracerebral (IC) inoculation of 0.02 ml of amoebae were done using insulin syringe (2). The same amount of distilled water was inoculated to nasal of mice for control. The mice monitored on daily observation and as soon as they present any brain involvement they were sacrificed. The brain of all animals was then dislocated and passaged in non-nutrient agar.

## Results

**Fig. 1:**
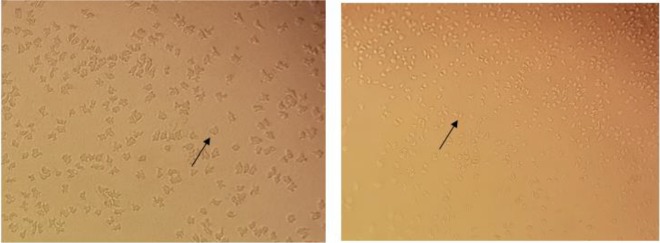
Brain culture of the infected mice with *N. australiensis* after few hours of culture

One mice out of seven infected mice showed clinical symptoms of meningoencephalitis similar to primary amoebic meningoencephalitis (PAM) due to *Naegleria fowleri* after 6 d of inoculation (Table 1). The infected mice showed behavioral changes including restlessness, turning heads, fast-moving erratically, impatience and other clinical signs due to Primary amoebic meningoencephalitis. The brain of the infected mice was then dislocated and showed various local hemorrhage and necrosis. Interestingly, within few hours of culture of the brain, many vegetative forms of amoebae were detected in non-nutrient plate culture (Fig. 1).

**Table 1:** Data of infected mice (BALB/c) with *N. australiensis* isolated from geothermal water sources

***Mice codes (BALB/c)***	***Presence of brain involvement symptoms***	***Culture of dislocated brain on non-nutrient agar plate***
Mice 1	-	-
Mice 2	-	-
Mice 3	-	-
Mice 4	+	+
Mice 5	-	-
Mice 6	-	-
Mice 7	-	-
^*^ Control mice	-	-

* Control mice was described as mice inoculated with distilled water

The amoebae showed wormy shape trophozoites with lobopodia mimicking vahlkampfiids amoebae and round double-walled cysts measuring 10–15 micron. The growth was fast and the plate covered with amoebae within few hours. The other infected animals and control mice showed no clinical symptoms until day 14. After 14 days all the animals sacrificed. The culture of brain in non-nutrient agar was negative up to two months.

## Discussion

The present study was the first to investigate the pathogenic potential of Iranian strain of *N. australiensis* in vivo in Iran. Only one mouse developed PAM after seven days of Intra-Nasal inoculation. The lack of brain involvement of other animals in the present study could be due to animal immune system or the amoebae did not reach to olfactory bulb of nostrils. So far, *N. australiensis* has been able to cause PAM in animals and also this strain has been isolated from brain of a fish (9).

*N. australiensis* has a lower pathogenic potential in comparison to *N. fowleri* (10). *N. australiensis* may lead to death of fewer animals and also these species need a longer incubation period for developing brain symptoms (11). The lower ability of pathogenic potential of *N. australiensis* may lead to negative virulence in animal models. To this end, the inoculation of 5000 *N. fowleri* amoebae to 44 mice, all mice died. However, the same amount of inoculation of *N. australiensis* to 44 mice showed that only 10 of them died (12, 13). In addition to *N. australiensis*, *N. italica* and *N. philippinensis* could cause PAM in mice (3–14). *N. australiensis* and *N. italica* kill experimental animals. However, no human infection due to these two other pathogenic *Naegleria* spp. has been diagnosed to date (15).

## Conclusion

Overall, the present research showed the pathogenic potential of *N. australiensis* isolated from environmental sources in Iran. More studies regarding the pathogenesis mechanisms of this species and the possibility of the disease development in human are of utmost importance.
